# Open and closed wedge osteotomes reduce the meniscus extrusion during walking with the knee adduction moment

**DOI:** 10.1002/jeo2.70795

**Published:** 2026-06-03

**Authors:** Yosuke Ishii, Akinori Nekomoto, Masakazu Ishikawa, Kyohei Nakata, Goki Kamei, Atsuo Nakamae, Yuko Nakashima, Shigeru Sato, Naoto Fujita, Nobuo Adachi

**Affiliations:** ^1^ Department of Bio‐Environmental Adaptation Sciences, Graduate School of Biomedical and Health Sciences Hiroshima University Hiroshima Japan; ^2^ Department of Orthopaedic Surgery, Graduate School of Biomedical and Health Sciences Hiroshima University Hiroshima Japan; ^3^ Department of Orthopaedic Surgery, Faculty of Medicine Kagawa University Kagawa Japan; ^4^ Translational Education and Research Center for Biomedical and Health Sciences Hiroshima University Hiroshima Japan

**Keywords:** dynamic meniscus, high tibial osteotomy, medial meniscus extrusion, open and closed wedged, walking

## Abstract

**Purpose:**

Medial meniscus extrusion (MME) is exaggerated by mechanical loading and exhibits a significant response during gait. Open‐wedge (OW) and closed‐wedge (CW) high tibial osteotomy (HTO) can reduce the mechanical loading associated with MME during walking; however, the impact of the differences in these surgical procedures remains unclear. This study aimed to compare MME behaviour during walking between OWHTO and CWHTO. It was hypothesised that CWHTO would demonstrate superior outcomes compared to OWHTO in terms of reducing mechanical stress.

**Methods:**

Twenty‐one knees from twenty‐one patients with knee osteoarthritis were enrolled and allocated to CW and OW groups. MME during walking was evaluated using ultrasonography synchronised with three‐dimensional motion analysis. The dynamic behaviour of MME was assessed by calculating the change between the MME value at initial contact and the maximum value observed during walking. Mechanical stress was evaluated using the knee adduction moment. These measurements were performed at two time points: preoperatively and postoperatively at approximately 24 months.

**Results:**

Both MME behaviour and knee adduction moment impulse values during walking were significantly reduced compared to baseline measurements MME behaviour (*p* = 0.0001) and knee adduction moment (*p* = 0.001), whereas no significant differences were observed between the groups.

**Conclusions:**

The differences between CWHTO and OWHTO were not detected in MME behaviour during walking.

**Level of Evidence:**

Level IV.

**Abbreviations**
CW: closed‐wedge
HKAA: hip‐knee‐ankle angle
HTO: high tibial osteotomy
JLCA: joint line convergence angle
KAM: knee adduction moment
KOOS: Knee Injury and Osteoarthritis Outcome Score
K/L: Kellgren–Lawrence
MCL: medial collateral ligament
MME: medial meniscus extrusion
MTPA: medial proximal tibial angle
OA: knee osteoarthritis
OW: open‐wedge
%MA: percentage of the mechanical axis
ΔMME: dynamic behaviour of medial meniscus extrusion

## INTRODUCTION

The meniscus absorbs load across the tibiofemoral joint through its hoop stress mechanism [25, 26]. The medial meniscus is frequently affected by pathological conditions such as degenerative tears, leading to medial meniscus extrusion (MME) with meniscal hoop function dysfunction [4, 5]. The MME gradually increases due to a meniscal behaviour with repetitive mechanical stress during daily living [7, 16, 20]. The MME behaviour is a key factor in knee osteoarthritis (OA), being closely associated with patient symptoms [3, 18] and disease progression [32]. Therefore, minimising MME behaviour is a promising option for patients with knee OA.

Varus malalignment increases the mechanical load on the medial tibiofemoral joint and has been associated with MME in several reports [10, 38]. High tibial osteotomy (HTO) redistributes this mechanical load [11] and has been shown to correct varus alignment [28] and reduce MME under both supine and standing conditions [1, 17]. Thus, HTO is expected to be a promising tool for addressing the MME under mechanical loads.

Among HTO techniques, closed‐wedge (CW) HTO has traditionally been used. In contrast, open‐wedge HTO (OWHTO) has also been established as a safe and time‐efficient procedure. However, OWHTO has been reported to increase the tibial slope [2, 29] and may induce medial collateral ligament (MCL) laxity because of the release technique [34, 36]. These biomechanical changes have been associated with increased MME [3, 24, 27], which may limit the effectiveness of HTO in reducing MME. In fact, a previous study comparing HTO techniques reported differences in postoperative MME responses assessed under supine conditions, demonstrating a favourable response by CWHTO [33]. Accordingly, CWHTO may have a more favourable effect on MME than OWHTO.

Notably, the MME underestimates static conditions in the supine and standing positions compared to those during walking [18, 22]. This may explain why mechanical stress during walking is two to three times greater than under static conditions [31]. In particular, the knee adduction moment (KAM) is a representative indicator of mechanical stress in the medial compartment of the knee and is correlated with MME behaviour during walking [20]. Therefore, assessing MME behaviour during walking may provide clinicians with valuable insights into the effects of HTO; however, these effects remain incompletely understood. This study aimed to investigate the effects of HTO techniques on MME behaviour during walking. Additionally, it was hypothesised that CWHTO would demonstrate superior outcomes compared to OWHTO in terms of reducing mechanical stress.

## MATERIAL AND METHODS

### Participants

Twenty‐five knees from 25 patients (10 females; mean age, 61.1 ± 7.5 years) diagnosed with primary unilateral or bilateral knee OA were recruited between April 2019 and June 2025 in this study. All the participants underwent HTO. Moreover, patients presenting with patellofemoral symptoms, based on physical examination findings, including radiographic evidence of OA and retropatellar crepitus, were preferentially selected for CWHTO. Participants were divided into two groups, OWHTO (OW group) and CWHTO (CW group).

The inclusion criteria for participants were as follows: (1) primary medial compartment OA radiographically, (2) hip‐knee‐ankle angle (HKAA) of <0°, (3) medial proximal tibial angle (MPTA) of <87° and (4) knee range of motion (ROM) greater than 120° without flexion contracture of <10°. The exclusion criteria were as follows: (1) use of walking aid; (2) lack of outpatient visits; (3) history of trauma, neurological disorder, or peripheral sensory disorder; (4) surgical complications related to infection and (5) combination techniques with pull‐out repair for medial meniscus posterior root tears. Consequently, twenty‐one knees from 21 participants were included in this study, and their demographic data are displayed in Table 1. All participants provided informed consent based on the study protocol, following approval of the protocol by the institutional review board.

**Table 1:** Demographic data of the participants.

	OW	CW	*p*‐values
*N*/Knees	11/11	10/10	
Sex (M:F)	6:5	4:6	
Age (years)	63.0 ± 6.4	62.5 ± 9.2	0.879
BMI (kg/m^2^)	25.9 ± 3.4	24.6 ± 3.8	0.272
FTA (deg)	180.2 ± 2.9	182.5 ± 3.8	0.160
K/L (I, II, III, IV)	1, 4, 4, 2	1, 5, 2, 2	
Meniscus tear			
Intact (%)	1 (9)	1 (10)	
Radial (%)	3 (27)	3 (30)	
Vertical (%)	1 (9)	1 (10)	
Horizontal (%)	1 (9)	1 (10)	
Degenerative (%)	5 (45)	4 (40)	

*Note*: Values are expressed as mean ± standard deviation.

Abbreviations: BMI, body mass index; CW, closed‐wedge high tibial osteotomy; FTA, femoral tibial angle; K/L, Kellgren–Lawrence; OW, open‐wedge high tibial osteotomy.

### Surgical procedure of HTO and arthroscopic evaluation of the meniscus

**Figure 1:**
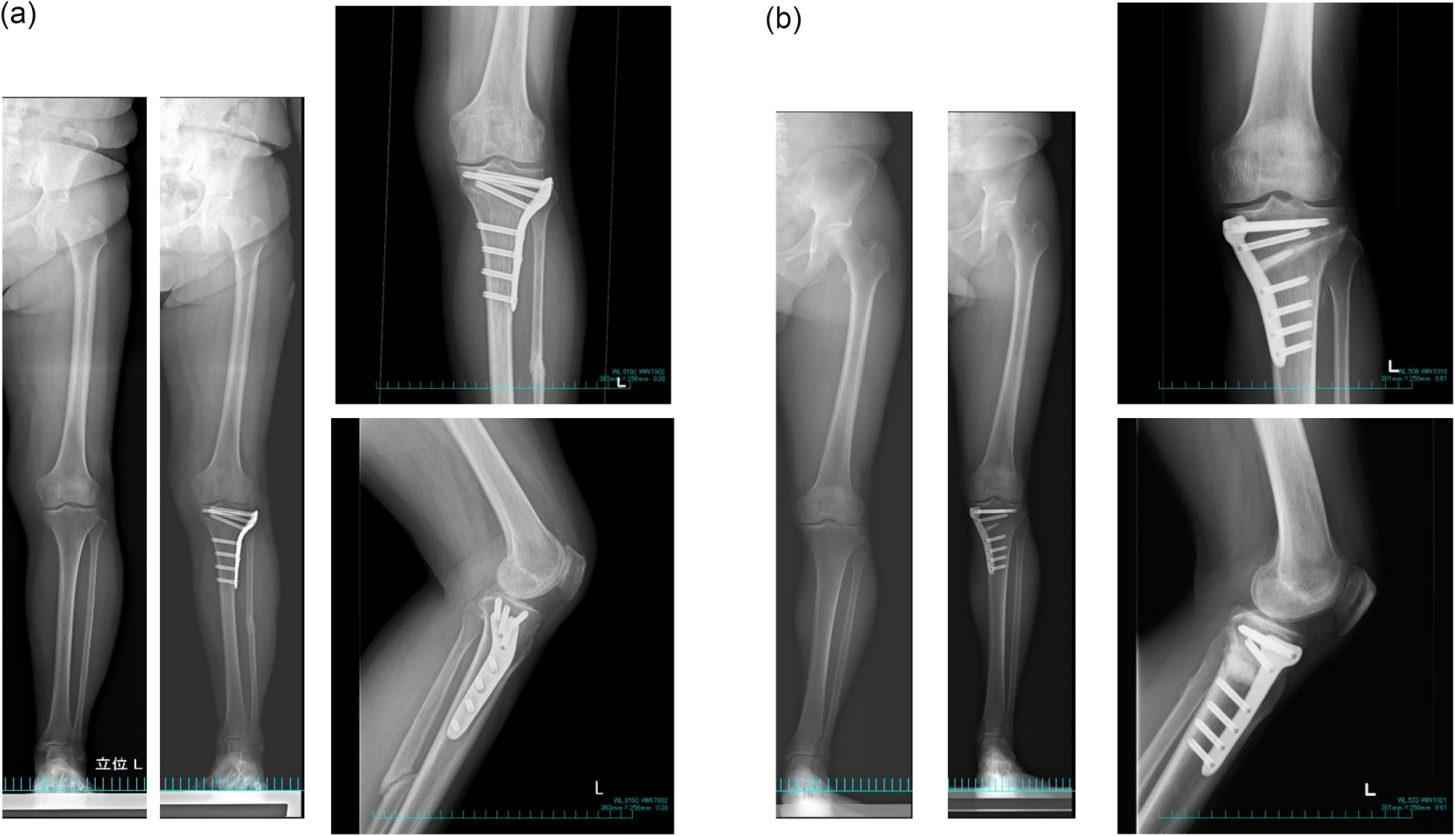
The type of high tibial osteotomy (HTO) techniques. The surgical techniques are illustrated as the closed‐wedge HTO (a) and open‐wedge HTO (b).

CWHTO was indicated for patients presenting with patellofemoral symptoms, as determined by physical findings involving radiographic OA changes and retropatellar crepitus. The HTO technique aimed to acquire and correct the varus malalignment by realigning the mechanical axis (MA) to pass through the lateral tibial eminence in weight‐bearing situations [30]. TriS® medial and lateral plates (Olympus Terumo Biomaterials Corp., Tokyo, Japan) were used to stabilise the corrected alignment. In the medial OWHTO, the bone substitute was implanted according to the gap size (Osferion 60®, Olympus Terumo Biomaterials Corp., Tokyo, Japan) (Figure 1). Moreover, arthroscopic evaluation of the meniscus was also performed. The meniscal condition and tear type were recorded.

During postoperative rehabilitation, 48 h after the procedure, range of motion exercises were initiated following the removal of the suction drain. Weight‐bearing training was started gradually, and partial weight‐bearing was performed 1 week postoperatively. Full weight‐bearing was performed 3 weeks postoperatively. During outpatient follow‐up, the patient's bone condition and postoperative infection were carefully monitored using radiological and clinical findings. Eventually, these procedures were demonstrated to mitigate complications such as unstable lateral hinge fractures during the union of the anterior flange.

### Assessments of low limb alignment

All participants were evaluated for knee disease severity and lower limb alignment. Kellgren–Lawrence (K/L) grades were assigned based on the assessment of the severity of knee OA using the radiographic image from the Rosenberg view. Lower limb alignment was evaluated by measuring varus knee alignment using radiographic images under weight‐bearing and full limb conditions. Based on knee alignment, which has been demonstrated to correlate with MME in a previous study [10], the HKAA, percentage of the MA (%MA), MTPA, and joint line convergence angle (JLCA) were assessed. These evaluations exhibited high reliability when performed by a single orthopaedic surgeon [17].

### Gait analyses

Walking kinematics and kinetics were evaluated using a system of 16 Vicon Motion cameras (100 Hz) and eight AMTI force plates (1000 Hz). Reflective markers were positioned on specific lower limb landmarks following the plug‐in‐gait lower body marker set protocol. The participants performed two walking task trials at a comfortable speed. Based on the ground reaction force over a threshold of 10 N, the heel contact to toe‐off was identified. The single‐stance phase was defined as the period between heel contact and toe‐off. The knee joint angles and moments during this phase were computed using Nexus 1.8.5, with all data normalised to 100 points. The primary focus of the analysis was frontal‐plane knee motion, including varus angles and peak KAM. The KAMs were quantified by measuring the peak value and impulse during the stance phase of a single gait cycle. Moreover, spatiotemporal data were included alongside gait speed measurements. These calculation processes were demonstrated using MATLAB R2020a (MathWorks).

### Evaluation of meniscus extrusion

**Figure 2:**
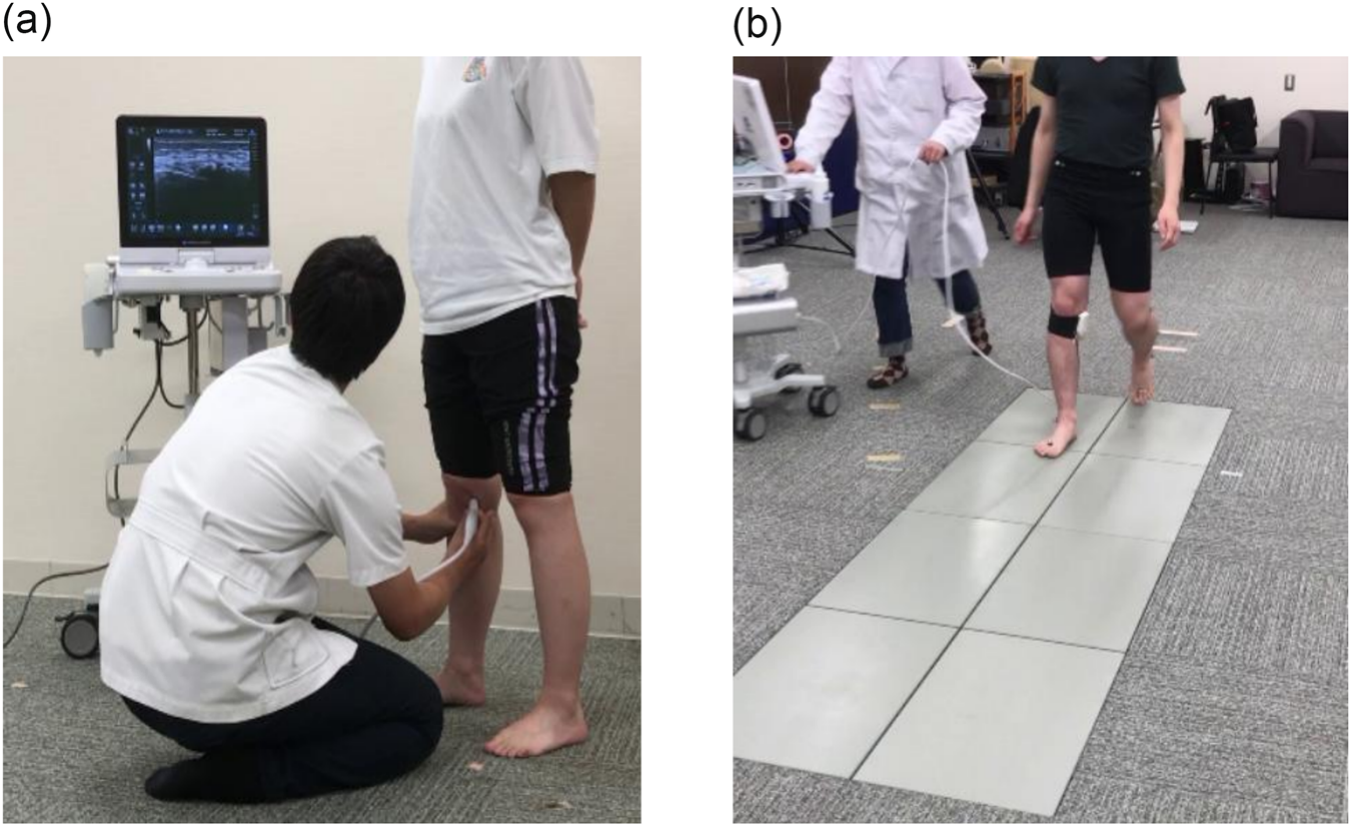
The evaluation of meniscus extrusion. A representative image of the medial meniscus after high tibial osteotomy. Static conditions during standing (a) and dynamic conditions during walking (b).

The medial meniscus was assessed using ultrasound with a unique prototype transducer of a linear array at 3–11 MHz (SNiBLE; KONICA MINOLTA). A longitudinal transducer was attached to the medial joint space, and a triangular morphological image of the medial meniscus within the tibiofemoral joint was obtained. The landmark was identified as the clearest image, delineating the boundary between the edge of the medial meniscus and the deep MCL. Static data were acquired in the supine and standing positions using the examiner's handle (Figure 2a).

**Figure 3:**
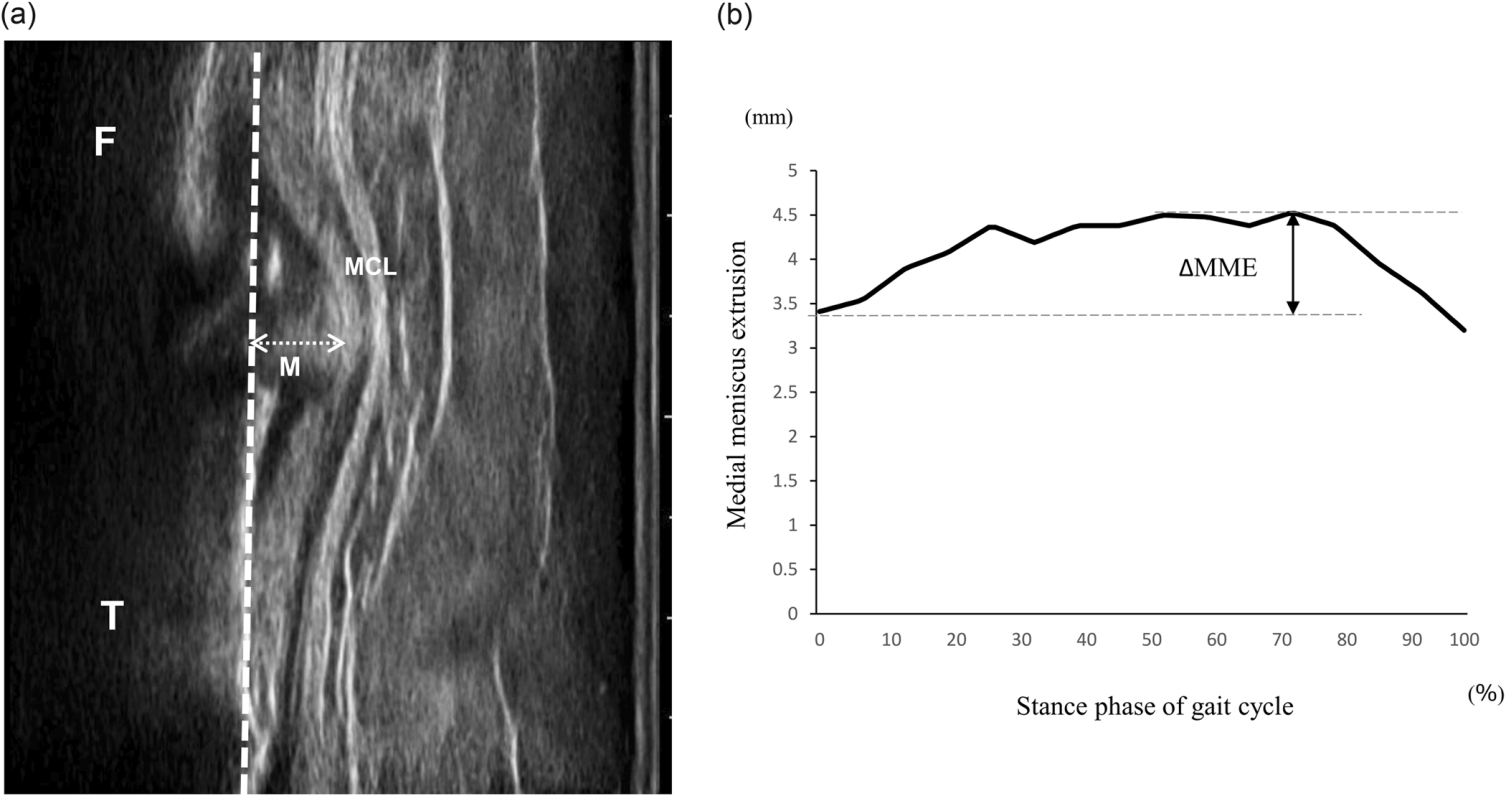
The calculation of the medial meniscus extrusion (MME) and its behaviour (ΔMME). A representative image of the medial meniscus. The dotted line and arrow indicate the tibial extension line, and the distance of meniscal displacement as the MME value. F, femora; M, meniscus; MCL, medial collateral ligament; T, tibia (a). The MME waveform during the stance phase of a single gait cycle was constructed from a sequential series of MME values. The double arrow shows as ΔMME, and it is calculated as the difference between the maximum and minimum MME values during walking. The dotted lines represent the maximum and minimum MME during walking (b). ΔMME, behaviour of medial meniscus extrusion.

During walking, a flexible specialised band was used to secure the transducer, allowing the patients to move their knee freely. The ultrasound device was synchronised with motion analysis using the electrocardiogram method. The patient's meniscus was visualised using the video mode of ultrasonography (Figure 2b). The analysis range was defined as the duration of the stance phase in a single gait cycle. Approximately 25 images extracted from the ultrasound video in a single trial were obtained, and the MME was measured in each image. Based on a previous study, the MME was determined as the distance from the cortex line of the tibial plate to the outermost edge of the meniscus using the Kinovea software (Figure 3a). Moreover, a waveform of the MME was generated by plotting continuous MME values recorded during walking. The ΔMME represented the dynamic behaviour of MME and was defined as the difference between the MME value at initial contact and the maximum value observed during walking, excluding the final three images. To compare the different durations across patients or trials, the data were standardised to 101 data points (Figure 3b). These structured procedures proved to be highly reliable [20].

### Clinical outcome assessment

Clinical evaluation was performed using the knee injury and osteoarthritis outcome score (KOOS). The KOOS, which comprehensively evaluates the health of the knee [35], includes five subscales: symptoms, pain, activity of daily living, sports/recreation and quality of life. All subscales were assessed for each patient at follow‐ups.

### Follow‐up and data processing

These measurements were performed at two time points: preoperatively and postoperatively at approximately 24 months. All outcomes were calculated as the change in values between the preoperative and postoperative assessments.

### Statistical analysis

All data were analysed using the Shapiro–Wilk test to confirm the normality of the data. Two‐way factorial analysis of variance with replication was performed to investigate the effect of follow‐up time and surgical technique on MME, clinical score, walking data and knee alignments. Bonferroni correction was performed for multiple comparison tests. Pearson or Spearman correlation coefficients were used to investigate the correlation in the change of ΔMME with the magnitude of change in mechanical stress values. The significance level was set at 5% and all statistical analyses were performed using SPSS version 22 (IBM Corp., USAIBM, US).

## RESULTS

### Participants' demographic data

No significant differences were observed in the demographic data. For meniscus quality, both groups demonstrated the most frequent degenerative tears (Table 1). Moreover, four patients presented with posterior root tears (OW: *n* = 2, CW: *n* = 2).

### Clinical and anatomical changes

Among the clinical and anatomical parameters, only a significant effect of time was observed (*p* < 0.01), with no group effect or interaction.

In both groups, all postoperative KOOS values significantly improved compared to baseline scores (Table 2).

**Table 2:** Comparison of changes in clinical parameters.

	OW			CW		
	Preoperative	Postoperative	*p*‐value [95% CI]	Preoperative	Postoperative	*p*‐value [95% CI]
KOOS‐s (%)	61.4 ± 10.1	75.1 ± 14.5	0.019 [2.56−24.7]	55.4 ± 11.5	80.1 ± 10.3	0.0001 [13.0−36.3]
KOOS‐p (%)	56.0 ± 11.1	77.8 ± 17.5	0.001 [10.1−33.5]	47.7 ± 14.9	85.3 ± 10.3	0.0001 [25.2−49.8]
KOOS‐a (%)	76.5 ± 7.7*	92.0 ± 9.9	0.001 [7.4−23.4]	64.5 ± 13.0	90.0 ± 9.1	0.0001 [17.1−33.8]
KOOS‐sf (%)	42.3 ± 14.5	63.6 ± 26.6	0.028 [2.5−40.1]	30.6 ± 25.6	61.1 ± 29.6	0.004 [10.7−50.2]
KOOS‐q (%)	29.3 ± 13.1	56.8 ± 25.3	0.001 [13.3−41.7]	26.7 ± 13.7	61.2 ± 22.7	0.0001 [19.6−49.4]

*Note*: Values are expressed as means ± standard deviation. The *p*‐values were shown with 95 Confidence interval [95% CI]. *Significantly higher than that observed in the CWHTO group preoperatively (*p* < 0.05).

Abbreviations: CI, confidence interval; KOOS: Knee Injury and Osteoarthritis Outcome Score; KOOS‐a, activities of daily living; KOOS‐p, pain; KOOS‐q, quality of life; KOOS‐s, symptoms; KOOS‐sf, sports/recreation.

Moreover, postoperative knee alignments, including the HKAA, %MA and MTPA, were significantly higher than those at baseline. Conversely, in the OW group, the postoperative JLCA was significantly lower than that at baseline (Table 3).

**Table 3:** Comparison of the changes in knee alignment.

	OW			CW		
	Preoperative	Postoperative	*p*‐value [95% CI]	Preoperative	Postoperative	*p*‐value [95% CI]
HKAA (deg)	−6.4 ± 3.2	1.0 ± 2.9	0.0001 [4.8−9.9]	−7.4 ± 3.2	−0.57 ± 2.7	0.0001 [4.2−9.4]
%MA (%)	24.0 ± 12.1	55.3 ± 10.1	0.0001 [25.3−37.1]	19.9 ± 10.2	48.0 ± 8.4	0.0001 [21.9−34.3]
MTPA (deg)	84.5 ± 2.6	90.6 ± 2.3	0.0001 [4.1−8.1]	83.2 ± 2.2	91.4 ± 2.0	0.0001 [6.1−10.3]
JLCA (deg)	2.5 ± 1.4	1.6 ± 1.1	0.001 [0.4−1.4]	3.1 ± 1.6	2.7 ± 1.5	0.132 [−0.1, 0.9]

*Note*: The values are expressed as means ± standard deviation. The *p‐*values were shown with 95 Confidence interval (95% CI).

Abbreviations: HKAA, hip‐knee angle; JLCA, joint line convergence angle; %MA, percentage of mechanical axis; MTPA, medial tibial plateau angle.

No significant difference was noted in the improvement in knee alignment without JLCA, and KOOS scores between the groups (Tables 2 and 3).

### Kinetics and kinematics change during walking

Among the kinetic and kinematic parameters, only a significant effect of time was observed (*p* < 0.01), with no group effect or interaction.

**Figure 4:**
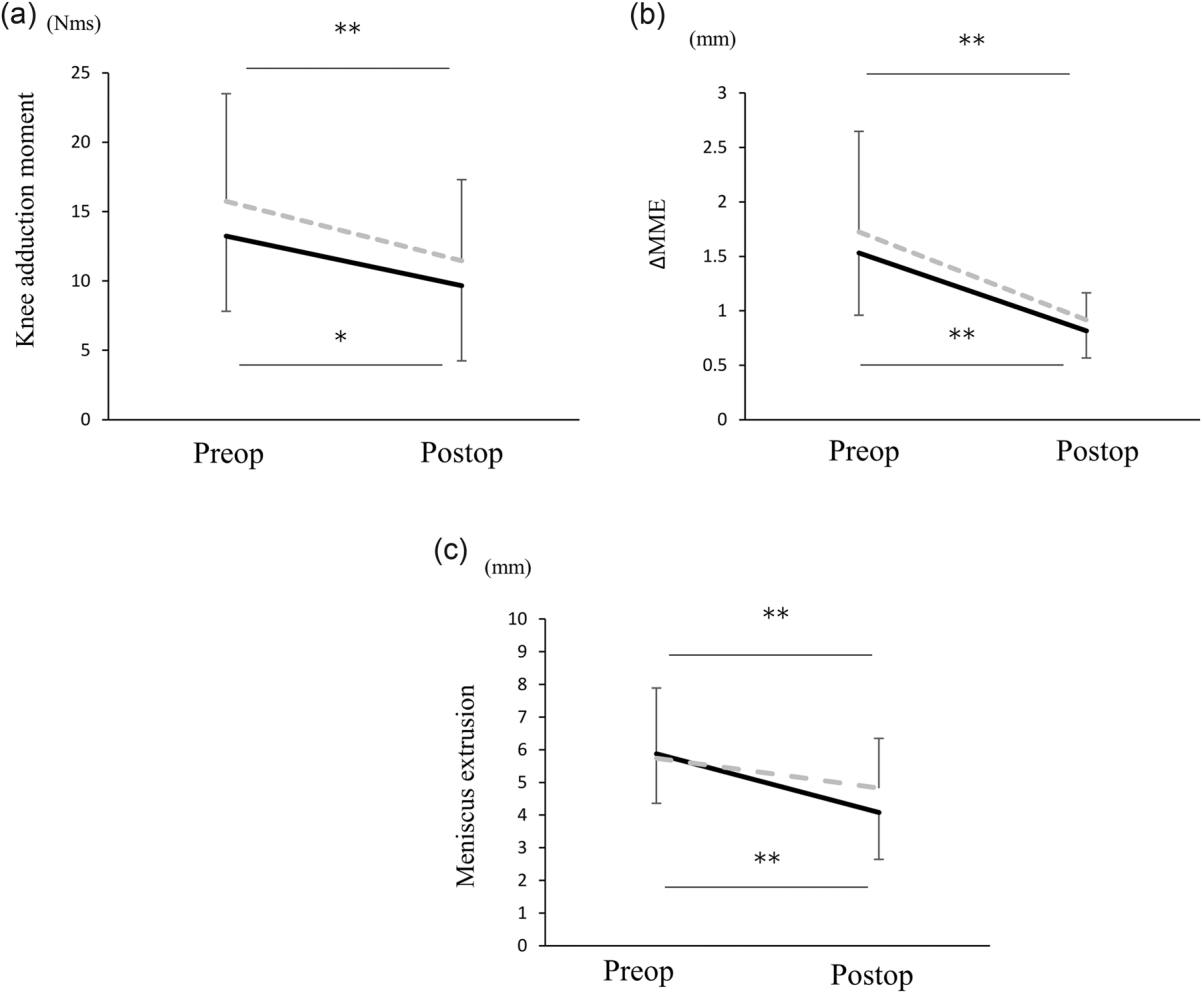
The comparison of the effect of high tibial osteotomy (HTO) on the meniscus parameters. Effect of high tibial osteotomy on meniscal parameters. The impulse of knee adduction moment (a), meniscal behaviour during walking (b) and standing (c). The lines with and without a dot display a closed‐wedge HTO and an open‐wedge HTO, respectively. preope: preoperative, postope: postoperative, ΔMME: behaviour of medial meniscus extrusion. * and ** Were shown <0.05, <0.01, respectively.

In both groups, postoperative varus angle, second KAM and impulse were significantly reduced (Figure 4a). No significant changes in the kinetics or kinematics were observed between the groups.

With regard to gait speed, only the CW group improved postoperatively when compared with that at baseline, but there was no significant difference between groups (Table 4).

**Table 4:** Comparison of kinetic and kinematic parameters during walking.

	OW			CW		
	Preoperative	Postoperative	*p*‐value [95% CI]	Preoperative	Postoperative	*p*‐value [95% CI]
Maximum varus angle (deg)	5.7 ± 3.8	0.9 ± 3.6	0.001 [2.2−7.3]	3.5 ± 3.4	−0.2 ± 4.6	0.009 [1.0−6.3]
First KAM (Nm/kg)	0.48 ± 0.14	0.40 ± 0.17	0.214 [−0.2, 0.04]	0.55 ± 0.17	0.51 ± 0.13	0.483 [−0.17, 0.08]
Second KAM (Nm/kg)	0.42 ± 0.15	0.30 ± 0.15	0.002 [0.05−0.1]	0.50 ± 0.17	0.37 ± 0.14	0.004 [0.04−0.1]
KAM impulse (Nms)	13.2 ± 5.4	9.7 ± 5.4	0.018 [0.6−6.4]	15.7 ± 7.8	11.5 ± 5.9	0.008 [1.2−7.3]
Walking speed (m/s)	0.91 ± 0.13	0.91 ± 0.10	0.992 [−0.08, 0.08]	0.83 ± 0.14	0.93 ± 0.16	0.025 [0.01−0.1]

*Note*: The values are expressed as means ± standard deviation. The *p*‐values were shown with 95 Confidence interval [95% CI].

Abbreviations: CW, closed‐wedge high tibial osteotomy; KAM, knee adduction moment; OW, open‐wedge high tibial osteotomy.

### Meniscus extrusions and their dynamics

Among the MME parameters, only a significant effect of time was observed (*p* < 0.001), with no group effect or interaction. In particular, ΔMME showed a significant main effect of time (*F* (1,19) = 21.53, *p* = 0.001, partial *η* ^2^ = 0.531). The mean reduction in ΔMME was 0.755 (95% CI, 0.414–1.095). However, no significant effect of group and its interaction was observed (group effect: *F* (1,19) = 0.61, *p* = 0.445, partial *η* ^2^ = 0.031; interaction (*F* (1,19) = 0.08, *p* = 0.783, partial *η* ^2^ = 0.004).

In the postoperative period, the minimum and maximum MME values were significantly reduced compared with those at baseline. Moreover, the ΔMME was also reduced compared to baseline. However, no significant differences were observed between the groups. (Figure 4b,c; Table 5).

**Table 5:** The comparison of MME parameters.

	OW			CW		
	Preoperative	Postoperative	*p*‐value [95% CI]	Preoperative	Postoperative	*p*‐value [95% CI]
Static condition						
Supine MME (mm)	5.2 ± 1.5	4.0 ± 1.5	0.001 [0.7−1.6]	5.1 ± 1.9	4.4 ± 1.6	0.004 [0.2−1.2]
Standing MME (mm)	5.9 ± 1.5	4.1 ± 1.4	0.0001 [1.1−2.3]	5.7 ± 2.2	4.8 ± 1.5	0.006 [0.2−1.5]
Walking condition						
Minimum MME (mm)	4.5 ± 1.9	3.0 ± 1.5	0.003 [0.6−2.5]	5.4 ± 2.3	4.1 ± 1.3	0.012 [0.3−2.2]
Maximum MME (mm)	6.0 ± 2.2	3.8 ± 1.6	0.001 [2.2−7.3]	7.1 ± 3.0	5.0 ± 1.5	0.002 [1.0−6.3]
ΔMME (mm)	1.5 ± 0.	0.8 ± 0.2	0.005 [0.2−1.1]	1.7 ± 0.9	0.9 ± 0.2	0.003 [0.3−1.2]

*Note*: Values are expressed as mean ± standard deviation. The *p*‐values were shown with 95 Confidence interval [95% CI].

Abbreviations: CW, closed‐wedge high tibial osteotomy; MME, medial meniscus extrusion; ΔMME, behaviour of medial meniscus extrusion; OW, open‐wedge high tibial osteotomy.

### The correlation between postoperative changes in the meniscus

The reduction in ΔMME was significantly correlated with KAM impulse (*r* = 0.45, *p* = 0.037). However, no significant correlations were observed for other parameters, including knee alignment and biomechanics.

## DISCUSSION

The present study demonstrated that HTO consistently reduced MME behaviour during gait. Furthermore, no significant differences were observed in MME values or KAM during gait between the HTO groups, in which the hypothesis regarding the effects of surgical techniques was not supported.

This finding indicates that HTO serves as a viable option for reducing MME despite the differences in surgical procedures. Previous studies have reported the impact of HTO procedures on KAM and identified no difference between OWHTO and CWHTO [6]. This outcome is consistent with present findings. MME is also known to be promoted by the laxity of soft tissues in the medial compartment of the knee, including the MCL and joint capsule [3, 27]. With OWHTO, immediate loosening of the MCL is observed following intraoperative release techniques [34, 36]. However, another study reported changes in laxity over time following OWHTO. It discovered an increase in laxity immediately after surgery; however, improvement was observed at the 12‐month follow‐up. No significant difference was observed between 12 and 24 months postoperatively, indicating that the MCL release technique had little influence [36]. In this study, follow‐up was performed for approximately 24 months after surgery, which may have allowed sufficient time for perimeniscal soft tissue healing. Therefore, these previous studies may elucidate the lack of differences in MME behaviour between OWHTO and CWHTO depending on the follow‐up period.

Present data confirmed that the decrease in ΔMME correlated only with KAM impulse and not with changes in static knee alignment. Deterioration of MME behaviour during gait is known to be caused by mechanical loading [19, 20], and a consistent association between MME behaviour and KAM has been demonstrated [13]. Notably, representative mechanical stresses, including KAM impulses, depend on variables such as ground reaction forces associated with gait dynamics and cannot be fully explained by static alignment alone. A previous study reported that static varus alignment only partially reflects the actual stress in the medial compartment [37]. These previous studies may explain the different correlations between the MME behaviour and static and dynamic mechanical loads. This highlights the importance of gait analysis assessment after HTO, in addition to static knee alignment, to manage the actual mechanical loads associated with MME.

Recent studies have reported a reduction in MME after HTO [15]. However, patients with MME often present with concomitant posterior root tears, and a certain proportion of such cases were observed in the present study. A cadaveric study examining the effect of HTO alone in patients with knee OA and concomitant posterior root tears demonstrated that, without additional intervention for the root lesion, HTO did not lead to a significant reduction in MME [14]. This finding suggests that the immediate mechanical effects of HTO on MME may be limited. One possible explanation for this discrepancy could be biological changes induced by sustained mechanical unloading. A recent study reported a shift in macrophage polarisation from pro‐inflammatory M1 to anti‐inflammatory M2 phenotypes following HTO, suggesting that the intra‐articular environment may change over time [39]. Therefore, HTO may contribute to improvements in MME not only through mechanical correction but also through longer‐term biological effects, although this interpretation is speculative and was not directly assessed in the present study. The association between KAM impulse and MME observed in this study may support the importance of mechanical factors, although further investigation is required.

An abnormal MME was defined as a value greater than 3 mm [8]. However, it was revealed that static MME did not achieve the normal MME value even after HTO, despite notable improvements in the clinical parameters. These factors frequently contribute to challenges in interpreting the clinical outcomes of patients with knee OA who underwent HTO. Some studies have demonstrated that MME behaviour was directly related to pain during walking, but MME during the resting state did not explain. Furthermore, a reduction in MME behaviour has been associated with improved clinical outcomes and decreased mechanical stress [21, 23]. Indeed, a significant decrease in ΔMME was also demonstrated in both groups. Therefore, these might emphasise that ΔMME should not be considered a surrogate for the MME values obtained supine and standing, but rather an independent parameter for determining the clinical effectiveness of HTO.

In this study, the relatively small sample size and exploratory design may raise concerns regarding statistical error in between‐group comparisons. However, the main effect of group demonstrated a small effect size (partial *η* ^2^ = 0.031), suggesting that the difference between surgical procedures was limited in magnitude. Furthermore, the reduction in ΔMME was approximately 40% in the OW group and 31% in the CW group, resulting in a between‐group difference of approximately 9%. In a previous study [21], a difference of approximately 25% in ΔMME reduction was observed between pain‐improved and nonimproved groups following lateral wedge insole intervention, which may represent a clinically meaningful change. Taken together, these findings may indicate that even if a statistically significant difference were detected with a larger sample size, the insufficient difference would likely remain from a clinical perspective. Although the present findings should be interpreted with caution due to the limited sample size and exploratory nature, given that based on that the primary objective of HTO is the improvement of clinical symptoms, the current results may provide supportive insight regarding the comparable reports of these procedures in future studies.

This study had some limitations. First, the small sample size limited the statistical power of the analyses and may have increased the risk of a type II error, potentially obscuring true differences between groups. In addition, the current sample size restricted the robustness of the findings and limited the generalisability of the results. It also prevented sufficient analysis of subgroups, including age, weight‐bearing status, knee severity and alignment. Second, the follow‐up period was approximately 24 months. This period was intended to determine the effect of HTO on the MME, which impacted the cumulative mechanical stress. However, the effects of HTO have been reported over a longer period than in our findings [9]. Then, the potential for long‐term follow‐up to reveal disparities in HTO efficacy cannot be excluded. Third, the present study excluded patients who underwent pull‐out or root repair, which may directly influence MME and clinical outcomes [12], thereby limiting our ability to assess responses to the combined surgical approach. Fourth, the applications of CW and OW HTO techniques are fundamentally different, it could not make direct comparison between the two approaches inherently difficult. Therefore, the findings of this study may have limited clinical applications. Future studies with a large sample size, a prolonged follow‐up period and detailed subgroup analyses of surgical techniques are needed.

## CONCLUSION

HTO can consistently reduce MME under mechanical loading during walking, although the differences were not detected between the OW and CW procedures.

## AUTHOR CONTRIBUTIONS

**Yosuke Ishii**: Acquisition and analysis of data; conception and design; drafting the article. **Akinori Nekomoto**: Recruiting the participants and analysis of data. **Masakazu Ishikawa**: Recruiting the participants and conception and design. **Kyohei Nakata**: Recruiting the participants. **Goki Kamei**: Recruiting the participants. **Atsuo Nakamae**: Recruiting the participants. **Yuko Nakashima**: Obtaining the ethical approval and the established method. **Shigeru Sato**: Analysis of data. **Naoto Fujita**: Interpretation of data. **Nobuo Adachi**: Final Approval of the manuscript.

## FUNDING INFORMATION

Japan Society for the Promotion of Science, Grant/Award Number: 23k16541.

## CONFLICTS OF INTEREST STATEMENT

Yosuke Ishii and Yuko Nakashima declare that they have the lendable device of ultrasound from KONICA MINOLTA, INC. The remaining authors declare that they have no conflict of interest.

## ETHICS STATEMENT

This study was approved by the Ethical Committee for Epidemiology of Hiroshima University, and informed consent was obtained from each participant (E2019‐1595).

## Data Availability

The datasets generated during and/or analysed during the current study are available from the corresponding author upon reasonable request.
